# Evaluating Tobacco Use Among United States Air Force Military Trainees: A Narrative Review of the Literature From 1998 to 2020

**DOI:** 10.7759/cureus.117320

**Published:** 2026-10-01

**Authors:** Parul Chhatpar, Mohit Chhatpar, Catherine Martsolf, Greg Doke, Nidhi Charan, Hythem Bishtawi

**Affiliations:** 1 Addiction Medicine, Hackensack Meridian Jersey Shore University Medical Center, Neptune, USA; 2 Addiction Medicine, Hartford Hospital, Hartford, USA; 3 Biology, Indiana University of Pennsylvania, Indiana, USA; 4 Family Medicine, Indiana Regional Medical Center, Indiana, USA; 5 Radiology, Cape Fear Valley Medical Center, Fayetteville, USA

**Keywords:** basic military training, military trainees, smoking initiation, smoking reinitiation, tobacco cessation, tobacco interventions, tobacco restrictions, united states air force

## Abstract

Tobacco use remains a health and readiness concern among military personnel. In the United States Air Force (USAF), mandatory tobacco abstinence during Basic Military Training (BMT) does not necessarily translate into sustained abstinence after training. This narrative review examines tobacco use among USAF trainees before and after BMT, factors associated with initiation and reinitiation, and prevention interventions during the transition to technical training.

PubMed was searched in 2024 for literature published from January 1998 through December 2020. Search terms addressed the Air Force and military training, tobacco and nicotine products, and prevention and cessation. One reviewer screened publications for eligibility, and 20 research studies were selected for narrative synthesis. Relevant literature on other military populations provided background. No new participants were enrolled, and no meta-analysis or formal risk-of-bias assessment was conducted.

The reviewed studies documented tobacco use before enlistment and initiation or reinitiation after mandatory abstinence. Findings highlighted prior tobacco use, intentions to use tobacco, peer influence, perceived harm, and access to products during technical training. Brief tobacco interventions improved some short-term beliefs, knowledge, and intentions, but the principal three-group trial did not demonstrate a significant difference in tobacco use between intervention groups at three months.

These historical findings support continued tobacco-prevention efforts beyond BMT, with attention to prior users and nonusers, multiple tobacco and nicotine products, social influences, and access both on and off base. Interpretation is limited by single-reviewer screening, the unavailable original executable search string, and the restriction to literature published through 2020.

## Introduction and background

Smoking among military personnel imposes health and readiness costs, including increased healthcare expenditure and lost productivity [1]. Military deployment has also been associated with smoking initiation and resumption [2]. Previous research has also shown that tobacco use in the military has been influenced by factors such as tobacco policies, product prices and availability, and tobacco-industry marketing toward service members [3-5]. These factors are especially important to consider among young recruits as they enter the structured environment of military training.

The United States Air Force (USAF) recruits are an important population for tobacco prevention as they enter military service. Basic Military Training (BMT) is the initial phase of enlisted training, during which recruits develop foundational military skills, discipline, and physical fitness. After BMT, enlisted airmen enter technical training, which provides specialized instruction and practical training for their assigned career field. Compared with the restricted BMT environment, technical training progressively restores personal independence and opportunities for off-base access. In a population-based assessment of 32,144 individuals entering USAF BMT, Haddock et al. reported that approximately 32% smoked cigarettes regularly before training. Smoking was associated with male sex, Euro-American race/ethnicity, lower educational attainment, alcohol use, binge drinking, smokeless-tobacco use, and lower physical activity [6]. These findings indicate that tobacco use in recruits can occur as part of a broader cluster of health-risk behaviors rather than as an isolated behavior.

Tobacco use among military trainees is not limited to cigarettes. Studies have also found smokeless tobacco use among active-duty military personnel [7]. Among USAF technical trainees, tobacco use before enlistment included cigarettes, hookah, cigarillos, smokeless tobacco, and e-cigarettes [8,9]. A study of 13,873 Air Force trainees also found that using two or more tobacco products was common, showing the importance of addressing more than just cigarette use in tobacco prevention efforts [10].

BMT provides an important opportunity to prevent tobacco use because all tobacco products are prohibited during training. This requires trainees to stop using tobacco for the duration of BMT. However, being required to stop using tobacco is different from choosing to quit, and previous USAF research has shown that many airmen return to tobacco use shortly after BMT [11,12]. Klesges et al. found that this tobacco-free period could help prevent relapse, while later studies examined ways to continue these prevention efforts during technical training [12-14].

The transition from BMT to technical training is an important time for tobacco prevention. Little et al. found that some airmen who had not previously smoked began using cigarettes, while some former smokers started smoking again during their first year of service [15]. Similar findings were reported for smokeless tobacco use [16]. Fahey et al. also found that many airmen began or returned to tobacco use during technical training. Access to these products varied, with e-cigarettes and some other tobacco products often obtained or first used at off-base locations [17].

Social and cultural factors can also influence tobacco use among airmen. Peer influence, stress, access to tobacco products, and attitudes toward tobacco use may affect whether someone chooses to use these products [3-5]. Research among Air Force personnel found that some airmen used e-cigarettes to cope with emotions or to feel more connected socially. The study also found that attitudes toward vaping and limitations in existing tobacco policies may contribute to e-cigarette use during technical training [18]. These findings identify social, emotional, and policy-related factors associated with e-cigarette use during technical training. They do not directly assess whether education about tobacco-related health risks prevents tobacco use.

Several interventions have attempted to capitalize on the period of mandatory abstinence. Brandon et al. described a trial designed to prevent initiation or relapse after 8.5 weeks of involuntary abstinence during BMT [13]. This duration reflects the historical training context of that study. Little et al. subsequently evaluated a Brief Tobacco Intervention (BTI) addressing multiple tobacco and nicotine products and targeting perceived harm and intentions to use tobacco [19]. The BTI was later evaluated during technical training in a cluster-randomized trial [14]. The intervention was based in part on the theory of planned behavior, which explains how attitudes, social influences, perceived control, and intentions can affect behavior [20]. Previous smoking cessation research has also shown that a person's intentions and perceived control can influence smoking behavior. However, the intention to quit does not always result in long-term abstinence [20].

Although previous research has documented tobacco initiation and reinitiation after BMT, questions remain about how to sustain tobacco abstinence during the transition to technical training [17]. Intervention studies have assessed different outcomes, including immediate changes in perceived harm and intentions and subsequent tobacco use, making it important to distinguish changes in beliefs from changes in behavior [14,19]. A focused synthesis is needed to clarify which factors are associated with starting or returning to tobacco use after mandatory abstinence and what evidence supports interventions to prevent these outcomes across different tobacco and nicotine products. Accordingly, this review examines tobacco and nicotine product use among USAF trainees before and after BMT, with particular attention to the transition to technical training. It synthesizes evidence on factors associated with initiation and reinitiation, including prior tobacco use, product type, and social and environmental influences. It also evaluates tobacco prevention interventions, distinguishing improvements in attitudes and intentions from reductions in tobacco use at follow-up.

## Review

Review design and literature search

This narrative literature review examined tobacco use among USAF trainees before and after BMT. PubMed was searched in 2024 for literature published from January 1, 1998, through December 31, 2020. Search terms addressed the Air Force and military training, tobacco and nicotine products, and prevention-related topics, as summarized in Table 1. Reference lists of highly relevant publications were also reviewed to identify additional USAF-focused articles within the prespecified publication period. The original executable PubMed search string was not retained, limiting exact replication of the search.

**Table 1:** Literature search strategy.

Search element	Search strategy
Database	PubMed
Publication period	January 1998 through December 2020
Air force and military training terms	“United States Air Force,” “USAF,” “Air Force,” “recruit,” “trainee,” “Basic Military Training,” “BMT,” and “Technical Training”
Tobacco and nicotine terms	tobacco, smoking, cigarettes, smokeless tobacco, snus, cigars, cigarillos, hookah, e-cigarettes, vaping, and nicotine
Additional prevention and behavior terms	prevention, cessation, relapse, initiation, reinitiation, intervention, and tobacco ban
Additional search method	Reference lists of highly relevant publications were reviewed to identify additional USAF-focused articles within the prespecified publication period.

The inclusion and exclusion criteria used to select publications are summarized in Table 2. Reference lists of relevant publications were also reviewed to identify additional USAF-focused articles. The search was conducted in 2024.

**Table 2:** Inclusion and exclusion criteria.

Category	Criterion
Inclusion	Original research involving United States Air Force (USAF) recruits, basic military trainees, or technical trainees
Inclusion	Studies examining tobacco use
Inclusion	Studies examining risk factors associated with tobacco use
Inclusion	Studies examining tobacco relapse
Inclusion	Studies examining tobacco initiation or reinitiation
Inclusion	Studies examining the use of multiple tobacco products
Inclusion	Studies examining access to tobacco products
Inclusion	Studies examining tobacco-prevention or cessation programs
Inclusion	Studies involving the broader US military provided useful background information
Exclusion	Studies focused only on civilian populations without findings related to tobacco use in the military training environment
Exclusion	Repeated findings already reported in other publications, when considered as primary studies.

PubMed identified 432 records. After 119 duplicate records were removed, 313 records were screened by title and abstract, and approximately 45 full-text articles were assessed for eligibility. Twenty research studies were selected for narrative synthesis. This count refers to included research studies, rather than the total number of references cited for background and context.

Study selection and data extraction

Publications were screened for eligibility by a single reviewer (Catherine Martsolf) using the inclusion and exclusion criteria defined in Table 2. These criteria included research involving USAF recruits or trainees that examined tobacco use, risk factors, relapse, initiation or reinitiation, multiple tobacco products, access, or prevention and cessation programs. Research involving other military populations was included when it provided relevant background.

For each publication, information on the study population, training stage, sample size, tobacco products, and main findings was reviewed. For intervention studies, the intervention and reported outcomes were also reviewed. Publications reporting similar airmen populations or related findings were discussed together. Repeated findings reported in other publications were not treated as separate primary findings. The findings were synthesized narratively. No meta-analysis or formal risk-of-bias assessment was conducted.

Recruitment, sample size, survey administration, and statistical analysis

No participants were recruited, and no primary data collection or statistical testing was undertaken for this narrative review.

Numerical results are attributed to the original studies. Frequencies and percentages, effect estimates, confidence intervals, and p-values are reported when available; missing frequencies were not reconstructed from rounded percentages.

Results

Pre-BMT Tobacco Use and Risk Factors

Haddock et al. surveyed 32,144 individuals entering the USAF and reported that approximately 32% had smoked cigarettes regularly before beginning BMT [6]. An additional 7.6% identified themselves as former smokers [6]. Smoking was more common among men, Euro-American trainees, and those with lower levels of education. Trainees who smoked were also more likely to report alcohol use, binge drinking, and smokeless tobacco use, while reporting lower levels of physical activity [6].

Later studies showed that the pre-enlistment tobacco profile had broadened beyond cigarettes. Little et al. documented cigarette, hookah, cigarillo, smokeless-tobacco, and e-cigarette use among USAF technical trainees [8]. Linde et al. examined smokeless tobacco use among 14,810 trainees and identified demographic and social correlates of use [9]. In a separate sample of 13,873 Air Force trainees, Little et al. found that dual- and poly-tobacco use were common and were associated with lower confidence in remaining tobacco-free, lower perceived harm, and tobacco-related social/environmental influences [10].

Relapse, Initiation, and Reinitiation After Mandatory Abstinence

The tobacco ban during BMT keeps trainees from using tobacco while they are there, but its effects do not always continue once BMT ends. Haddock et al. studied 4,303 airmen who returned to smoking after BMT and found that 3,003 (69.8%) smoked again within the first month. Most were still in a training environment when they had their first cigarette after BMT [11]. These findings suggest that the period immediately following BMT is an important time to focus on preventing a return to tobacco use.

Little et al. followed 2,188 USAF personnel during their first year of service and identified distinct predictors of cigarette initiation and reinitiation [15]. Among those without established smoking, ownership of cigarette-branded merchandise was the strongest reported predictor of initiation (odds ratio (OR): 3.81, 95% confidence interval (CI): 1.67-8.71). Among former smokers, intention to use tobacco was the strongest reported predictor of reinitiation (OR: 2.08, 95% CI: 1.53-2.83) [15]. A related longitudinal analysis showed that smokeless-tobacco initiation and reinitiation also occur after the BMT abstinence period, extending the concern beyond cigarettes [16].

Tobacco Use During Technical Training

Fahey et al. found that tobacco use often returned during technical training [17]. Before BMT, 1,161 airmen (39.4%) reported regularly using at least one tobacco product. At follow-up during technical training, the authors reported tobacco use in 552 airmen (21.3%). They separately reported 432 previous users (37.4%) who started using tobacco again and 116 airmen (6.4%) who had not regularly used tobacco before BMT but began using it during technical training [17]. E-cigarettes were reported most often, followed by cigarettes, smokeless tobacco, cigarillos or little cigars, cigars, hookah, and pipes [17].

Airmen obtained tobacco products varied by product type. Cigarettes and smokeless tobacco were obtained both on and off base, and were often first used in designated smoking areas. E-cigarettes, cigarillos or little cigars, and hookah were more commonly obtained off base and first used at places such as vape shops, hookah bars, or cigar lounges [17]. This suggests that tobacco policies on military installations may not be enough to limit access because trainees can still obtain these products off base.

Social and Behavioral Influences

Several factors were associated with airmen's use of tobacco, including their intentions to use tobacco, peer influence, beliefs about tobacco use, confidence in remaining tobacco-free, perceived health risks, and previous tobacco use [10,14-16,19]. One study found that several factors influenced e-cigarette use during technical training. Some airmen did not see a strong reason to avoid vaping, while others used e-cigarettes to cope with emotions or as a way to socialize with others. Participants also felt that existing tobacco policies did not fully address e-cigarette use [18]. Previous research has also shown that tobacco prices, availability, military culture, and tobacco-industry marketing have influenced tobacco use among military personnel [3-5].

Intervention Design and the Three Intervention Conditions

The historical evidence reviewed included a three-group cluster-randomized trial during technical training [14]. Airmen were randomized by squadron to one of three conditions: Brief Tobacco Intervention (BTI) plus the Airman's Guide to Remaining Tobacco Free (AG), AG alone, or the National Cancer Institute's Clearing the Air booklet (CTA) [14].

The trial approached 3,347 airmen; 2,999 (89.6%) consented, 2,969 (99.0% of consenting airmen) were eligible, and 2,611 (87.9% of eligible airmen) completed the three-month follow-up [14]. Participants were at least 18 years old and able to understand the English-language consent process. The BTI comprised a 40-minute group session addressing tobacco-related attitudes, intentions, norms, knowledge, and refusal self-efficacy, informed by motivational interviewing, the theory of planned behavior, and behavioral-economic principles [14,19,20]. The AG condition used a 46-page booklet tailored to airmen, and the CTA condition used a general smoking-cessation booklet; each included an approximately five-minute discussion [14].

The original trial evaluated tobacco use at three months using a generalized estimating equations logistic regression approach with covariate adjustment and multiple imputation for loss to follow-up [14]. The study did not demonstrate a statistically significant difference in tobacco use between the three intervention conditions at follow-up (p = 0.454) [14]. However, the BTI + AG condition produced favorable short-term changes in perceived harm, intentions to use tobacco, tobacco knowledge, and normative beliefs [14]. These findings were consistent with the earlier 1,055-participant evaluation of the BTI, which showed immediate improvements in several cognitive targets of tobacco prevention [19].

Discussion

Multiple Tobacco Products and Prevention Needs

The studies included in this review show that tobacco use among USAF trainees has changed over time. Earlier research found that many recruits had already smoked cigarettes before entering BMT [6]. Later studies found that trainees were also using smokeless tobacco, e-cigarettes, hookah, cigars, and cigarillos, with some using more than one type of tobacco product [8-10]. These findings are important because prevention programs that focus only on cigarette smoking may not address the other tobacco and nicotine products used by airmen.

Mandatory Abstinence and Sustained Cessation

The tobacco ban during BMT creates a period of mandatory abstinence, but the observed early return to tobacco use and initiation during technical training indicate that restrictions alone may not produce sustained cessation [11,17]. Temporarily removing access does not necessarily alter intentions, peer influences, or access after training. Interventions using this tobacco-free period have therefore attempted to support continued abstinence after restrictions end [12,13,21].

It is also important to consider whether an airman used tobacco before BMT. Airmen who previously used tobacco may need support to prevent them from returning to use, especially when they experience cravings, peer influence, or regain access to tobacco products. Airmen who have never used tobacco may need prevention strategies focused on keeping them from starting. One study found that the intention to use tobacco was strongly associated with returning to cigarette use among former smokers, while owning cigarette-branded merchandise was associated with starting cigarette use [15]. Similar findings have also been reported for smokeless tobacco [16].

The Post-BMT Transition and Tobacco Access

Technical training may be an especially important time for tobacco prevention because airmen have more independence, meet new peers, and have more opportunities to access tobacco products. Some products, particularly e-cigarettes, were often obtained off base [17]. This means that policies controlling tobacco prices, sales, or smoking areas on military installations cannot completely prevent trainees from accessing tobacco elsewhere. Previous research has also shown that discounted tobacco products, product availability, and tobacco-industry marketing toward military personnel have played a role in tobacco use within the military [3-5].

Social factors may also influence tobacco use during technical training. Research on the use of multiple tobacco products found relationships between tobacco use and factors such as confidence in remaining tobacco-free, perceived harm, and social or environmental influences [10]. Another study found that some airmen used e-cigarettes to manage emotions or as a way to connect with others socially [18]. These findings suggest that prevention programs should address more than the health effects of tobacco. Peer influence, stress, social situations, and the environment surrounding airmen may also need to be considered.

Brief Interventions and Continued Support

Research on the BTI showed both positive results and limitations. Airmen who received the intervention showed short-term improvements in their knowledge about tobacco, beliefs about its harmful effects, attitudes toward tobacco use, and intentions to use tobacco [14,19]. However, these short-term changes did not necessarily lead airmen to remain tobacco-free over time. The theory of planned behavior helps explain how attitudes, social influences, perceived control, and intentions may affect tobacco use [20]. Previous smoking-cessation research has also found that a person's intentions and perceived control can influence whether they continue or stop smoking [20]. Although the BTI improved some tobacco-related beliefs, knowledge, and intentions, the trial did not find a statistically significant difference in tobacco use between intervention groups at the three-month follow-up [14]. This suggests that one brief intervention may not be enough to prevent tobacco use when airmen continue to experience stress, peer influence, and access to tobacco products.

More support throughout technical training may help airmen remain tobacco-free. Follow-up sessions, continued educational materials, peer support, and efforts to address tobacco access both on and off base could be considered [14,17]. Tailored tobacco-control programs have also been evaluated in military populations [21]. These findings support further evaluation of sustained, military-specific prevention approaches.

Tobacco prevention may also be more effective when addressed alongside other health behaviors. Among recruits, smoking has been associated with alcohol use, binge drinking, lower levels of physical activity, and the use of other tobacco products [6]. Research has documented the health and productivity costs of smoking among USAF personnel [1] and changes in tobacco use during military deployment [22]. Because these behaviors may occur together, tobacco prevention programs could also include stress management, responsible alcohol use, physical fitness, sleep, and other healthy behaviors.

Limitations and Future Research

This review has several limitations. The studies included were published over more than two decades, and tobacco products and military tobacco policies changed during that time. Many of the studies also relied on participants to report their own tobacco use, which may have affected the accuracy of the results. In addition, some of the publications came from related USAF research projects and may have included similar groups of participants. Article screening was performed by a single reviewer without independent duplicate screening, which may have increased the risk of selection bias. The original executable PubMed search strategy was unavailable, limiting reproducibility. No formal risk-of-bias assessment or meta-analysis was conducted. Finally, this review only included literature published through 2020. Future research should include more recent studies and examine ways to help airmen remain tobacco-free throughout their first year of military service.

## Conclusions

Although tobacco use is not permitted during BMT, it continues to be a concern among USAF trainees. The tobacco ban prevents use during BMT, but some airmen begin using tobacco or return to previous use once they enter technical training.

Tobacco prevention efforts should continue after BMT and address different tobacco and nicotine products, previous tobacco use, peer influence, stress, and access to tobacco products both on and off base. Continued support during technical training may help more airmen remain tobacco-free.
